# Supplementary material for: Rationale and Emerging Evidence on the Potential Role of HoLEP-Mediated Relief of Bladder Outlet Obstruction in NMIBC Outcomes Through Optimal Management of Chronic Urinary Retention
Source: Cancers (Basel). 2025 Dec 1;17(23):3864. doi: 10.3390/cancers17233864 (PMC12691192; doi:10.3390/cancers17233864)
Supplement: Supplementary file 1 [file cancers-17-03864-s001.zip › cancers-3966291-supplementary.pdf]

Supplementary Table S1. Search strategy, inclusion, and exclusion criteria used in the narrative review.

Domain	Description
**Databases searched**	PubMed/MEDLINE, Embase, and Scopus were systematically queried for English-language studies published between January 2000 and October 2025.
**Keywords and search terms**	Search strings combined controlled vocabulary (MeSH/Emtree) and free-text terms covering four domains:<br>Population: "bladder cancer," "urothelial carcinoma," "non–muscle-invasive bladder cancer," "NMIBC," "Ta," "T1," "carcinoma in situ."<br>Exposure/functional domain: "chronic urinary retention," "postvoid residual," "PVR," "lower urinary tract symptoms," "LUTS," "bladder outlet obstruction," "BOO," "benign prostatic hyperplasia," "BPH."<br>Interventions: "Holmium laser enucleation of the prostate," "HoLEP," "transurethral resection of the prostate," "TURP," "endoscopic enucleation," "open/simple prostatectomy."<br>Outcomes/therapy: "recurrence," "progression," "intravesical therapy," "BCG," "mitomycin C," "response," "Qmax," "IPSS," "quality of life."
**Search filters**	Human studies only; English language; adult populations (≥18 years). Reference lists of key reviews, EAU and IBCG guidelines, and recent mechanistic studies were manually screened to identify additional eligible publications.
**Review process**	Two independent reviewers screened titles and abstracts. Full texts were reviewed for eligibility, and disagreements were resolved by consensus or third-reviewer adjudication. The search and selection process followed a PRISMA-inspired flow (see Supplementary Figure 1).
**Inclusion criteria**	Studies were eligible if they:<br>– Enrolled adults diagnosed with NMIBC or included cohorts with BOO/BPH/urinary retention relevant to NMIBC;<br>– Reported at least one oncologic outcome (recurrence, progression, response to intravesical therapy) or functional endpoint (postvoid residual, Qmax, IPSS, catheter independence, peri-operative safety);<br>– Quantitatively evaluated BOO or urinary retention (e.g., PVR thresholds, LUTS/IPSS scores) or described BOO interventions (HoLEP, TURP, endoscopic or open prostatectomy).*
**Exclusion criteria**	Excluded were:<br>– Non-peer-reviewed reports or conference abstracts without full data;<br>– Animal or purely in vitro studies not directly linked to NMIBC-relevant mechanisms;<br>– Narrative commentaries, letters, or reviews without extractable data;<br>– Publications lacking clear outcome reporting on recurrence, progression, or functional results.*
**Data extraction and synthesis**	Data were abstracted on study design, sample size, patient/tumor characteristics, BOO or retention measures (PVR, LUTS/IPSS), intervention type, follow-up duration, and primary oncologic and functional outcomes. Because of study heterogeneity, results were summarized thematically across five predefined domains: (1) pathophysiology of retention-related carcinogenesis, (2) prognostic significance of BOO/PVR, (3) interaction with intravesical therapy, (4) effects of BOO correction—particularly HoLEP, and (5) comparative outcomes versus TURP.
**Quality appraisal**	Each study was qualitatively assessed for risk of selection bias, misclassification of BOO/retention, confounding (e.g., tumor risk or treatment history), and outcome ascertainment variability. The appraisal was descriptive and aligned with SANRA guidelines rather than formal quantitative scoring; a concise synthesis appears in Supplementary Table 2.

Supplementary Table S2. Risk of Bias Assessment Using an Adapted ROBINS-I Framework for Non-randomized Studies Evaluating BOO/Retention and NMIBC Outcomes.

Ref.	Confounding	Selection of Participants	Classification of Exposure/Intervention	Deviations from Intended Interventions	Missing Data	Outcome Measurement	Selective Reporting	Overall ROBINS-I Judgment	Key Reasons
[21] Sazuka et al.	Serious — Limited adjustment for tumor biology and IVT heterogeneity	Moderate — Single center; unclear criteria for PVR testing	Moderate — PVR measured but no standardized schedule	Moderate — IVT not uniform	Moderate — Some loss to follow-up	Moderate — Recurrence assessed variably	Moderate	Serious	Retrospective, non-standard PVR measurement, no risk-stratified HRs
[3] Lunney et al.	Serious — Subjective LUTS-based BOO measure; residual confounding likely	Moderate	Serious — BOO classification via LUTS categories only	Low	Moderate	Moderate	Moderate	Serious	Symptom-based BOO surrogate; limited progression data
[4] Di Gianfrancesco et al.	Serious — PVR threshold not validated; differential PVR measurement	Moderate	Moderate	Moderate	Moderate	Moderate	Moderate	Serious	Multicenter heterogeneity in IVT and cystoscopy schedules

[5] Can et al.	Serious — Remodeling metrics not standardized; confounding by duration of BOO	Moderate	Moderate	Low	Moderate	Moderate	Moderate	Serious	BOO not quantified consistently; progression data limited
[2] NMIBC + LUTS/BOO cohort	Serious — Variable PVR cutoffs; mixed-risk population	Moderate	Moderate	Moderate	Moderate	Moderate	Moderate	Serious	IVT heterogeneous; limited survival outcomes
[23] Garg et al.	Critical — Allocation to HoLEP vs TURP vs no surgery highly confounded	Serious — Non-standard, preference-based selection	Moderate	Moderate	Moderate	Moderate	Moderate	Critical	Severe confounding by indication; no multivariable oncologic modeling

[8,9, 25,26,29] HoLEP functional series	Moderate — BOO well characterized but not cancer populations	Low–Moderate	Low — Clear surgical modality classification	Low	Moderate	Low–Moderate	Moderate	Moderate	Large, high-quality functional data; limited oncologic relevance

Rating scale:
Low = comparable to a high-quality randomized study;
Moderate = sound for a non-randomized study but with some concerns;
Serious = important problems likely to affect validity;
Critical = severe bias that substantially limits interpretability;
NI = no information.

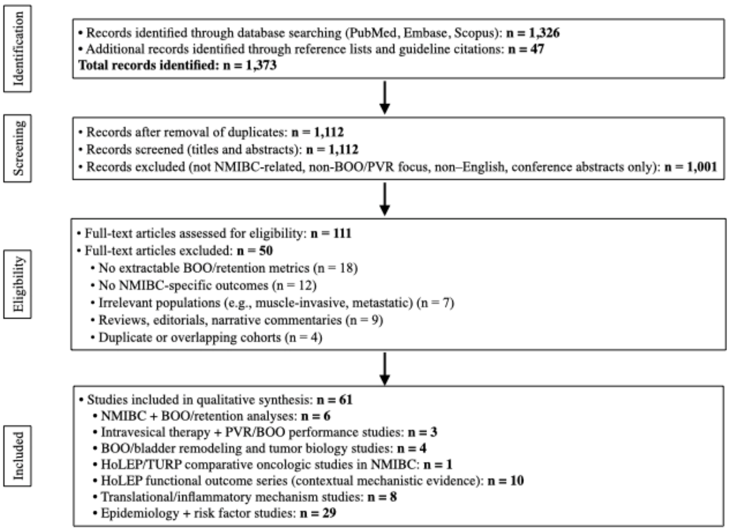

Supplementary Figure S1. PRISMA-Inspired Flow Diagram of Study Identification and Selection.

Supplementary Figure S1 presents the PRISMA-inspired flow of study identification, screening, eligibility assessment, and final inclusion for this narrative synthesis. The figure adheres to PRISMA 2020 conceptual structure but is adapted for a narrative review without formal quantitative synthesis.
